# Toxic Effects of Aflatoxin B_1_ in Chinese Sea Bass (*Lateolabrax maculatus*)

**DOI:** 10.3390/toxins13120844

**Published:** 2021-11-26

**Authors:** Kai Peng, Bing Chen, Hongxia Zhao, Wen Huang

**Affiliations:** Institute of Animal Science, Guangdong Academy of Agricultural Sciences, Key Laboratory of Animal Nutrition and Feed Science in South China, Ministry of Agriculture and Rural Affairs, Guangdong Provincial Key Laboratory of Animal Breeding and Nutrition, Guangzhou 510640, China; chenbing114@163.com (B.C.); zhaohongxia8866@163.com (H.Z.); huangwen549@126.com (W.H.)

**Keywords:** aflatoxin B_1_, toxicity, *Lateolabrax maculatus*

## Abstract

This study was performed to assess the effects of dietary aflatoxin B_1_ (AFB_1_) on the growth, antioxidant and immune response, digestive enzyme activities, and intestinal morphology of *Lateolabrax maculatus* during a 56-day feeding trial. Four diets were formulated including 0, 0.1, 0.5, and 1.0 mg/kg of AFB_1_. Each diet was randomly assigned to 3 fish tanks with 40 fish per tank. Results indicated that the fish’s final body weight, weight gain rate, specific growth rate, feed intake, condition factor, viscerosomatic index, hepatosomatic index, and intestinesomatic index decreased (*p* < 0.01) as dietary AFB_1_ increased. AFB_1_ levels in diets increased (*p* < 0.05) serum total antioxidant capacity (TAOC), superoxide (SOD), catalase, malondialdehyde (MDA), alkaline phosphatase (AKP), and lysozyme (LZM), and increased (*p* < 0.05) the TAOC, SOD, MDA, AKP, LZM, and immunoglobulin M in the livers of the fish. Dietary AFB_1_ decreased (*p* < 0.05) intestinal trypsin activity and induced intestinal injury. In summary, dietary AFB_1_ up to 1.0 mg/kg was toxic to *L. maculatus* as judged by reduced growth, enhanced antioxidant and immune response, decreased intestinal trypsin activity, and impaired intestinal morphology.

## 1. Introduction

Aflatoxin B_1_ (AFB_1_) is mainly produced by *Aspergillus flavus,* which exists in some raw feed materials for animal use, such as maize, peanut, and wheat flour [1]. AFB_1_ is a major challenge to aquaculture due to its high toxicity to aquatic animals and great threat to food safety. The biotransformation of AFB_1_ mainly occurs in the liver and intestine [2]. Intake may induce an inflammatory response, interrupt intestinal integrity, and eventually inhibit the growth of fish [3,4]. At present, research in this field has focused mainly on the effects of dietary AFB_1_ on growth, bioaccumulation, muscle quality, immune response, hematology, and hepatic function indices of aquatic animals [5,6,7,8,9,10,11,12,13,14,15,16,17,18]. Little information is available that evaluates the effect of AFB_1_ on the health of the gut, the largest digestive and immune organ in the animal body. Strengthening research in this area can provide a better understanding of the mechanism for AFB_1_-induced changes in the growth and intestinal health of aquatic animals.

The biological effects AFB_1_ has on aquatic animals are directly associated with the animal species and the dietary concentrations of AFB_1_ [5]. The effects of AFB_1_ on the growth and health of tilapia (*Oreochromis niloticus* × *O. aureus*) [6], grass carp (*Ctenopharyngodon idella*) [7], common carp (*Cyprinus carpio*) [8], channel catfish [9], *Clarias batrachus* [10], rainbow trout (*Oncorhynchus mykiss*) [4,11], *Sciaenos ocedatas* [12], *Litopanaeus vannamei* [13,14,15], turbot (*Scophthalmus maximus*) [16], gibel carp [17], and Indian carp (*Labeo rohita*) [18] have been assessed. However, information is rare regarding *Lateolabrax maculatus*, a popular carnivorous fish in the southern region of China in recent years, known for its rapid growth and superb taste [19]. In 2020, the nationwide fish yield exceeded 160,000 tons, according to the 2020 China Fishery Statistical Yearbook. Previous studies documented that *Litopanaeus vannamei* and gibel carp (*Carassius auratus gibelio*) could tolerate dietary AFB_1_ up to 2 mg/kg [13] and 0.5 mg/kg [17], respectively. Other species tolerated dietary AFB_1_ ranging from 0.1 to 2.5 mg/kg [6,7,9]. This had not yet been evaluated for *L. maculatus,* however. The present study was conducted to assess the effects of dietary AFB_1_ on growth, antioxidant and immune response, digestive enzyme activities, and intestinal morphology of *L. maculatus*.

## 2. Results

### 2.1. Growth Performance

Increasing dietary AFB_1_ from 0 to 1.0 mg/kg linearly and quadratically decreased (*p* < 0.01) the final body weight (FBW), weight gain rate (WGR), specific growth rate (SGR), and feed intake (FI), but did not alter the feed coefficient (FC) or survival rate (SR) of the fish (*p* > 0.05) (Table 1). The condition factor (CF), viscerosomatic index (VSI), and intestinesomatic index (ISI) linearly decreased (*p* < 0.05), and the hepatosomatic index (HIS) linearly and quadratically decreased (*p* < 0.05) as dietary AFB_1_ increased.

### 2.2. Antioxidant and Immune Response

The serum total antioxidant capacity (TAOC), superoxide dismutase (SOD), and catalase (CAT) linearly increased (*p* < 0.05), and malondialdehyde (MDA) linearly and quadratically increased (*p* < 0.01) as dietary AFB_1_ increased and reached significance at the level of 1.0 mg/kg (Table 2).

Increased dietary AFB_1_ linearly and quadratically increased (*p* < 0.05) serum alkaline phosphatase (AKP) and linearly increased (*p* < 0.05) lysozyme (LZM). The serum glutathione peroxidase (GPx) was similar among groups (*p* > 0.05).

The liver TAOC and MDA were linearly increased (*p* < 0.05), and SOD was linearly and quadratically increased (*p* < 0.05) as dietary AFB_1_ increased.

Increased dietary AFB_1_ linearly increased (*p* < 0.05) liver AKP, LZM, and immunoglobulin M (IgM). Dietary treatment did not alter the CAT, GPx, or complement C3 (C3) in the liver of fish (*p* > 0.05).

### 2.3. Intestinal Digestive Enzyme Activities and Histological Appearance

**Figure 1:**
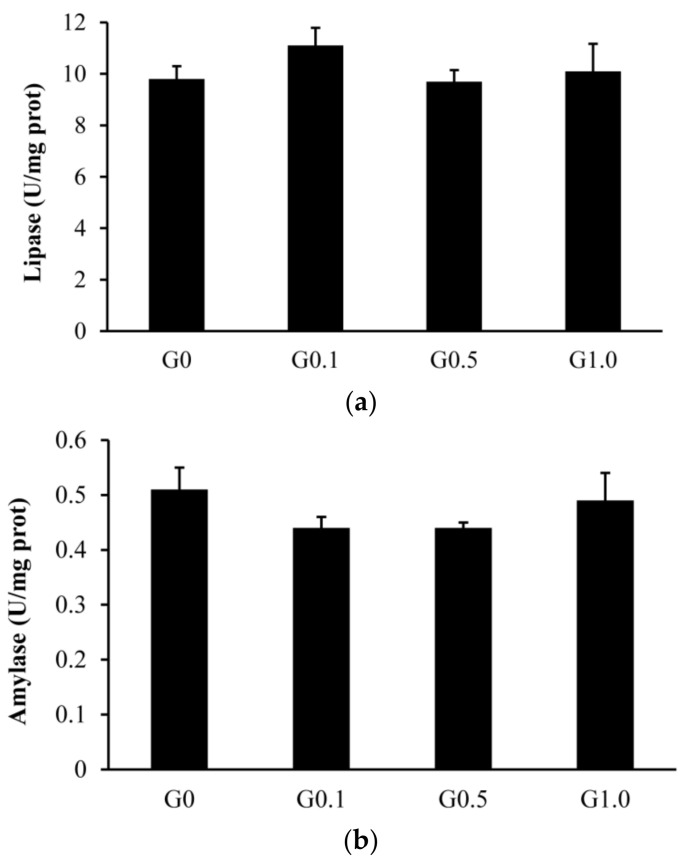

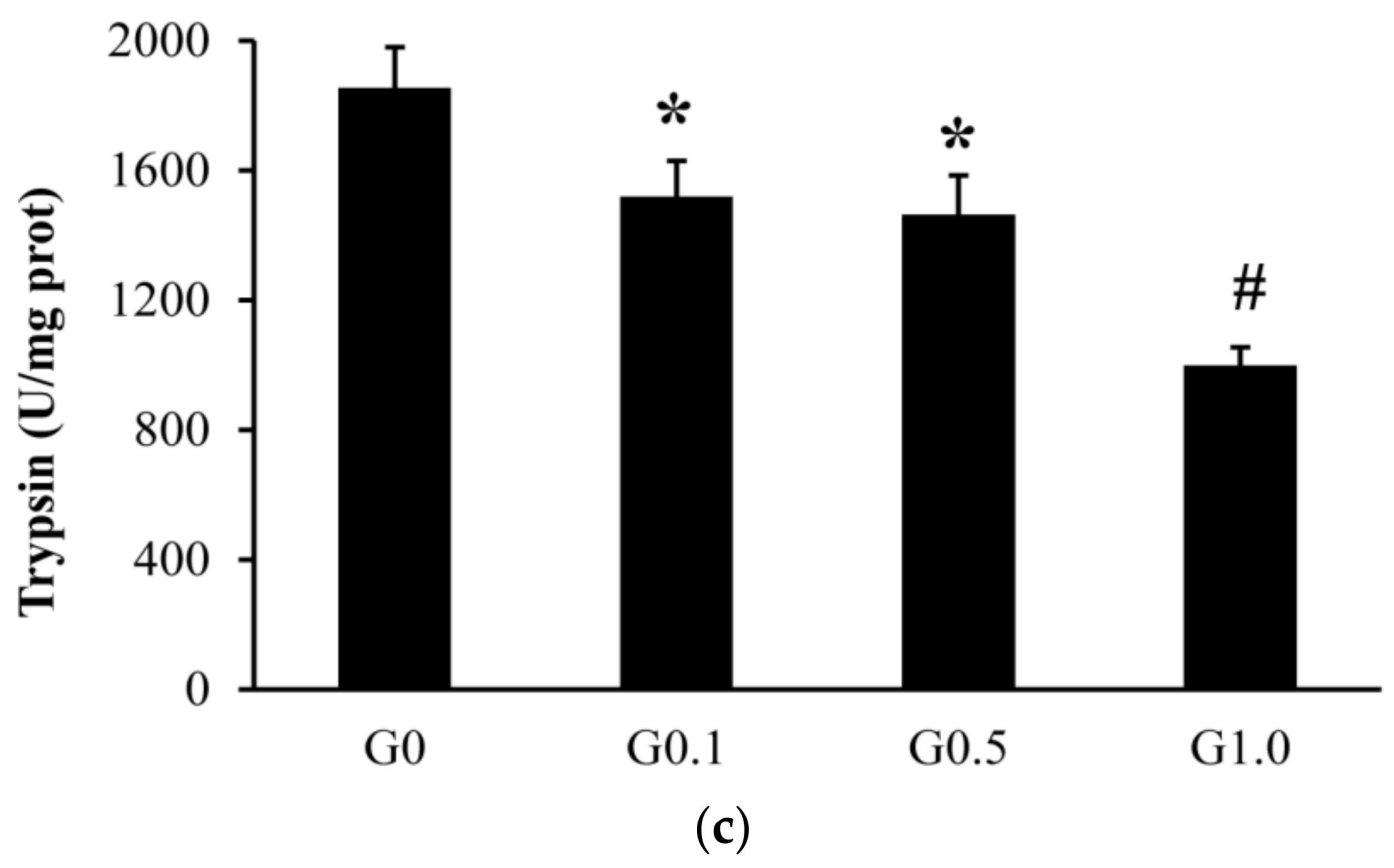
Effects of AFB_1_ on the intestinal digestive enzyme activities ((**a**), lipase; (**b**), amylase; (**c**), trypsin) of *L. maculatus*. G0–G1.0, basal diet added 0, 0.1, 0.5 and1.0 mg/kg of AFB_1_. * *p* < 0.05 compared to G0. ^#^ *p* < 0.05 compared to G0.1.

The intestinal trypsin activity was lower (*p* < 0.05) in G0.1 and G0.5 than in G0, and was lower (*p* < 0.05) in G1.0 than in other groups (Figure 1). The activities of lipase and amylase were similar among groups (*p* > 0.05).

**Figure 2:**
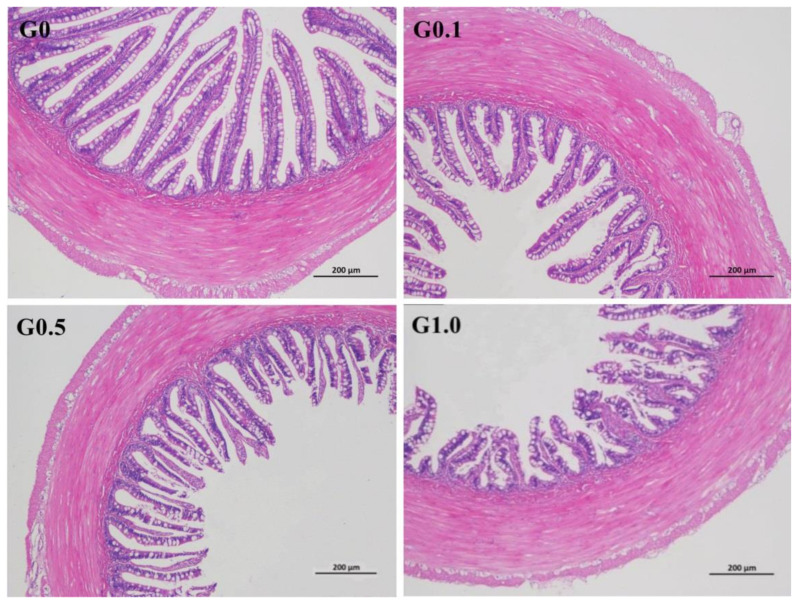
Effects of AFB_1_ on the intestinal histological appearance (×100) of *L. maculatus*. G0–G1.0, basal diet added 0, 0.1, 0.5 and 1.0 mg/kg of AFB_1_.

The intestinal villus in G0 was most regular in shape (Figure 2), whereas the villus in G0.1, G0.5, and G1.0 had different degrees of deformation as reflected by the irregular arrangement of the villus.

## 3. Discussion

### 3.1. Effect of AFB_1_ on the Growth of L. maculatus

The similar SR of fish among the groups suggested that dietary AFB_1_ up to 1.0 mg/kg did not alter the survival of *L. maculatus*. However, the decreased FI, WGR, and SGR as dietary AFB_1_ increased suggests that AFB_1_ adversely impacts the palatability of feed and the growth of *L. maculatus*. To the best of our knowledge, this is the first study assessing the effects of dietary AFB_1_ on the growth of *L. maculatus*. Similar results were also reported in tilapia [6], grass carp [7], common carp [8], *Pelteobagrus fulvidraco* [20], channel catfish [9], *Clarias batrachus* [10], rainbow trout [11], *Litopanaeus vannamei* [13], and gibel carp [17]. Growth inhibition is regarded as one of the main toxic effects of AFB_1_ on aquatic animals [1]. It has been reported that dietary AFB_1_ inhibits the growth of gibel carp by inducing liver function impairment and metabolic disorders [17]. In this study, the decreased hepatosomatic index, along with the declined intestinesomatic index of *L. maculatus* as dietary AFB_1_ increased, suggests that AFB_1_ may cause dysorganoplasia of the liver and intestine since AFB_1_ can induce degeneration and hepatocyte necrosis [21] and weaken the intestinal barrier function [22]. Using growth performance as the evaluation index, the recommended inclusion level of AFB_1_ in the *L. maculatus* diet is less than 1.0 mg/kg.

### 3.2. Effects of AFB_1_ on the Antioxidant and Immune Response of L. maculatus

The assessment of serum antioxidant and immune parameters can provide a better understanding of the mechanism for AFB_1_-induced damage in the growth and health of *L. maculatus*. In this study, the increased TAOC in either serum or the liver indicated that dietary AFB_1_ up to 1.0 mg/kg enhanced the antioxidant response of *L. maculatus*. Antioxidant enzymes including SOD and CAT have been known to play a key role in alleviating oxidative stress via scavenging reactive oxygen species [23]. The increased SOD and CAT activities, along with increased MDA concentrations in fish fed AFB_1_-treated diets in this study, suggest that dietary AFB_1_ causes oxidative stress. It has been reported that SOD could catalyze the dismutation of superoxide anion free radicals and thereby alleviate cellular DNA damage [24]. CAT protects the cell from oxidative injury by catalyzing hydrogen peroxide decomposition [25]. MDA is a product of lipid peroxidation, which can induce oxidative stress [26]. These increased antioxidant parameters in fish fed AFB_1_-treated diets are most likely in response to the physiological toxicity or oxidative stress stimulated by AFB_1_ rather than an improved antioxidant capacity of the fish. Similar results were also reported by Wang et al. [14], stating that including 5 mg/kg of AFB_1_ in *Litopenaeus vannamei* diets induced dysregulation of the antioxidant system of shrimp by increasing SOD and CAT activities and MDA concentration during the 30 days of the AFB_1_ challenge.

Similarly, the increased immune parameters as reflected by the increased AKP and LZM in the serum, as well as the increased AKP, LZM, and IgM in the livers of the fish, suggests that dietary AFB_1_ enhanced the immune response of *L. maculatus*. AKP, an unconventional immune protein, influences inflammation through the regulation of purinergic signaling [27]. LZM, a critical defense protein in the innate immune system, plays an important role in defending against microbial invasion [28]. IgM is the first antibody to respond to an antigen and is an effective defense factor against adverse stress [29]. Similar results were reported by Li [30], stating that dietary inclusion of 10 mg/kg and 50 mg/kg of AFB_1_ increased serum LZM and AKP activities and IgM concentration in *Cyprinus* carp. 

### 3.3. Effects of AFB_1_ on Intestinal Digestive Enzyme Activities and the Histological Appearance of L. maculatus

Intestinal digestive enzymes, including trypsin, lipase, and amylase, are often used as indicators to assess the digestive process of fish [31] or as feedback related to changes in feed formula [32]. Although the effects of AFB_1_ on the intestinal digestion of livestock have been assessed [33,34], information is rarely available for fish. In this study, dietary AFB_1_ significantly decreased intestinal trypsin activity but did not alter the lipase and amylase activities of *L. maculatus*. Wang et al. [35] reported that including 55 μg/kg of AFB_1_ in *Cyprinus carpio* diets decreased the apparent digestibility of crude protein owing to decreased intestinal trypsin activity. A similar result was observed by Ostrowski–Meissner [36], in that dietary AFB_1_ of up to 210 μg/kg only decreased the digestibility of crude protein in the intestines of ducklings. However, including 300 μg/kg of AFB_1_ in piglet diets [33] and 80 μg/kg of AFB_1_ in broiler diets [34] did not affect their intestinal digestive enzyme activities. Variations among studies seem to be attributed to species differences and may result from different concentrations of AFB_1_ in diets. In this study, the decreased growth of *L. maculatus* fed a diet containing 1.0 mg/kg of AFB_1_ may partly contribute to the decreased digestibility of crude protein, as reflected by the declined intestinal trypsin activity. However, crude protein digestibility was not evaluated in the present study and further research is still needed to confirm this hypothesis.

Changes in histological morphology commonly indicate pathological alteration caused by feed sources [37]. In this study, the atrophic intestinal villus observed in the AFB_1_-treated groups indicated that dietary AFB_1_ caused intestinal injury to *L. maculatus*. This is similar to the report by Wang et al. [38], stating that including 5 mg/kg of AFB_1_ in *Litopenaeus vannamei* diets destroyed the histomorphology of the intestine by reducing the height of the intestinal villus and completely detaching epithelial cells from the basement membrane. Others also observed similar results in rainbow trout (*Oncorhynchus mykiss*) [4] and common carp [8]. Documentation shows that such histological damage interferes with the absorption of nutrients [39,40]. In tilapia, the decreased growth performance of the fish was due to intestinal lesions induced by AFB_1_ [39]. The intestinal histological damages induced by AFB_1_ in this study may also account for the decreased growth performance of *L. maculatus*.

## 4. Conclusions

Dietary AFB_1_ up to 1.0 mg/kg enhanced antioxidant and immune response, decreased intestinal trypsin activity, and induced intestinal histological damages in *L. maculatus*, eventually reducing the growth performance of the fish. The findings of this study provide a better understanding of the mechanism for AFB_1_-induced damages in the growth and intestinal health of *L. maculatus*.

## 5. Materials and Methods

### 5.1. Experimental Diets

The compositions of the experimental diets are shown in Table 3. Four diets were prepared including 0 (G0), 0.1 (G0.1), 0.5 (G0.5), and 1.0 (G1.0) mg/kg of AFB_1_ (from *Aspergillus flavus*, Sigma, Canada). Dietary AFB_1_ concentrations were determined using liquid chromatography tandem mass spectrometry [41]. The actual AFB_1_ concentrations in G0, G0.1, G0.5, and G1.0 were 0, 0.09, 0.47, and 1.02 mg/kg, respectively.

### 5.2. Feeding Trial

In total, 480 juvenile *L. maculatus* (initial body weight 2.9 ± 0.02 g) were randomly distributed into 12 tanks (40 fish per tank), with 3 tanks per diet. Fish were hand-fed to apparent satiation twice daily at 07:00 and 19:00 for 56 days. During the feeding trial, the water temperature was 25–27 °C, dissolved oxygen was above 6.0 mg/L, pH was 7.4–8.0, and ammonia and nitrite levels were below 0.01 mg/L. The protocol of this study was approved by the Animal Care and Use Committee of Guangdong Academy of Agricultural Sciences (Guangzhou, China).

### 5.3. Sampling

At the termination of the trial, fish were fasted for 24 h and then anesthetized with 3-aminobenzoic acid ethyl ester methanesulfonate (40 mg/L, Sigma, Oakland, CA, USA) before sampling. Fish per tank were counted and weighed to analyze the SR, FBW, WGR, SGR, and FC. Feed intake (FI) was determined as the gravimetric difference between the feed offered and orts. Six fish in each tank were randomly selected for analysis of CF, VSI, his, and ISI.

Blood was collected from the caudal veins of six fish in each tank, kept at 25 °C for 30 min, and centrifuged at 8000× *g* for 10 min. Serum was stored at −80 °C for subsequent analysis of serum antioxidant and immune indexes. 

The livers of three fish per tank were taken to determine antioxidant and immune indexes. 

The intestines of three fish per tank were sampled for trypsin analysis (Ultraviolet colorimetry), lipase (colorimetry), and amylase (colorimetry) activities using commercial kits supplied by Nanjing Jiancheng Bioengineering Institute (Nanjing, China). The intestines of three other fish per tank were randomly collected for intestinal histological examination [42].

### 5.4. Sample Analyses

Proximate compositions of diets, including dry matter, crude protein, crude lipid, and ash, were measured following the AOAC method [43].

The TAOC (colorimetry), SOD (hydroxylamine method), CAT (ammonium molybdate spectrophotometric method), GPx (colorimetry), MDA (thiobarbituric acid method), AKP (microplate culture method), and LZM (turbidimetry) in the serum and liver were determined using commercial kits provided by Nanjing Jiancheng Bioengineering Institute (Nanjing, China). Concentrations of liver IgM and complement C3 were determined by ELISA kits using immunoturbidimetry (R&D Systems, Minneapolis, MN, USA).

### 5.5. Calculations and Statistical Analysis

The WGR, FI, FC, SR, SGR, VSI, his, and ISI were calculated by Peng et al. [44].

All data were analyzed by ANOVA and the SAS Mixed procedure system [45] with a tank as the statistical unit. Polynomial contrasts were used to analyze the linear and/or quadratic responses to dietary AFB_1_ concentrations. Differences were compared using LSMEANS with the PDIFF option and adjusted with a Tukey test. Significance was regarded as *p* < 0.05.

## Figures and Tables

**Table 1:** Effects of AFB_1_ on survival and growth of *L. maculatus*.

Items ^3^	Diets ^1^				SEM	P	Polynomial Contrasts ^2^	
	G0	G0.1	G0.5	G1.0			*L*	*Q*
SR, %	86.67	89.52	92.38	90.48	1.38	0.591	0.302	0.435
FBW, g	45.56 ^a^	45.31 ^a^	41.34 ^a^	26.70 ^b^	2.41	<0.001	<0.001	0.002
WGR, %	1464.43 ^a^	1455.98 ^a^	1323.00 ^a^	818.07 ^b^	82.76	<0.001	<0.001	0.001
SGR, %/d	4.10 ^a^	4.09 ^a^	3.96 ^a^	3.31 ^b^	0.10	<0.001	<0.001	<0.001
FI, g/fish	48.52 ^a^	46.54 ^a,b^	43.50 ^b^	27.83 ^c^	2.50	<0.001	<0.001	<0.001
FC	1.14	1.10	1.13	1.17	0.01	0.301	0.258	0.154
CF, g/cm^3^	1.65 ^a^	1.62 ^a,b^	1.56 ^b^	1.57 ^b^	0.01	0.036	0.007	0.581
VSI, %	9.27 ^a^	8.29 ^b^	7.55 ^c^	6.77 ^d^	0.15	<0.001	<0.001	0.633
HSI, %	0.84 ^a^	0.62 ^b^	0.54 ^b^	0.56 ^b^	0.03	0.001	0.001	0.039
ISI, %	0.63 ^a^	0.64 ^a^	0.51 ^b^	0.53 ^b^	0.01	0.004	0.040	0.148

^1^ G0–G1.0, basal diet added 0, 0.1, 0.5 and 1.0 mg/kg of AFB_1_. ^2^ *L*, linear effect; *Q*, quadratic effect. ^3^ SR, survival rate; FBW, final body weight; WGR, weight gain rate; SGR, specific growth rate; FI, feed intake; FC, feed coefficient; CF, condition factor; VSI, viscerosomatic index; HSI, hepatosomatic index; ISI, intestinesomatic index. ^a,b,c,d^ Different letters within a row denote difference (*p* < 0.05).

**Table 2:** Effects of AFB_1_ on the antioxidant and immune response of *L. maculatus*.

Items ^3^	Diets ^1^				SEM	P	Polynomial Contrasts ^2^	
	G0	G0.1	G0.5	G1.0			*L*	*Q*
Serum antioxidant								
TAOC, U/mL	4.12 ^b^	6.24 ^a,b^	5.91 ^a,b^	7.26 ^a^	0.46	0.082	0.023	0.609
SOD, U/mL	153.55 ^b^	163.96 ^a,b^	161.62 ^a,b^	171.97 ^a^	2.82	0.126	0.026	0.995
CAT, U/mL	11.55 ^b^	14.51 ^a,b^	15.36 ^a,b^	19.96 ^a^	1.28	0.115	0.025	0.707
GPx, U/mL	363.19	410.42	438.68	421.97	15.24	0.374	0.162	0.314
MDA, nmol/mL	3.68 ^b^	2.76 ^b^	3.32 ^b^	7.15 ^a^	0.58	0.003	0.003	0.004
Serum immune								
AKP, U/L	275.87 ^c^	283.95 ^c^	382.74 ^b^	507.73 ^a^	29.46	<0.001	<0.001	0.017
LZM, U/mL	308.00 ^b^	441.26 ^a^	471.37 ^a^	481.59 ^a^	25.46	0.023	0.012	0.108
Liver antioxidant								
TAOC, U/mg prot	0.37 ^b^	0.40 ^b^	0.66 ^a^	0.70 ^a^	0.07	0.148	0.038	0.941
SOD, U/mg prot	229.56 ^b^	264.53 ^a,b^	270.47 ^a,b^	310.43 ^a^	11.00	0.044	0.037	0.047
CAT, U/mg prot	47.82	46.60	50.57	49.78	1.85	0.903	0.614	0.960
GPx, U/mg prot	30.39	27.86	26.12	34.55	1.57	0.269	0.434	0.097
MDA, nmol/mg prot	0.70 ^b^	0.74 ^b^	1.94 ^a^	2.68 ^a^	0.28	0.002	<0.001	0.237
Liver immune								
AKP, U/mg prot	34.31 ^b^	40.57 ^b^	54.21 ^b^	92.78 ^a^	9.01	0.003	0.001	0.052
LZM, U/mg prot	10.34 ^b^	11.37 ^b^	16.29 ^b^	25.39 ^a^	2.01	0.004	0.001	0.097
IgM, μg/mg prot	31.18 ^b^	39.63 ^a,b^	48.99 ^a,b^	64.33 ^a^	5.44	0.153	0.019	0.239
C3, μg/mg prot	26.68	25.95	27.92	32.66	1.46	0.404	0.160	0.370

^1^ G0–G1.0, basal diet added 0, 0.1, 0.5 and 1.0 mg/kg of AFB_1_. ^2^ *L*, linear effect; *Q*, quadratic effect. ^3^ TAOC, total antioxidant capacity; SOD, superoxide dismutase; CAT, catalase; GPx, glutathione peroxidase; MDA, malondialdehyde; AKP, alkaline phosphatase; LZM, lysozyme; IgM, immunoglobulin M; C3, complement C3. ^a,b,c^ Different letters within a row denote difference (*p* < 0.05).

**Table 3:** Ingredients and proximate composition (g/kg DM) of the basal diet.

Ingredients	
Fish meal	180
Casein	180
Soy protein concentrate	160
High gluten	280
Monocalcium phosphate	15
Fish oil	40
Soybean oil	20
Soy lecithin	20
Vitamin premix ^1^	2
Mineral premix ^2^	5
Choline chloride	5
Vitamin C ester	1.5
Lysine	0.3
Methionine	2.2
Betaine	5
Zeolite powder	30
Cellulose	54
Condensed tannins	0
Proximate composition	
Dry matter	912.4
Crude protein	406.7
Crude lipid	109.8
Ash	73.0

^1^ One kilogram of diet provided: VA, 3230 IU; VD, 1600 IU; VE, 160 mg; VK_3_, 4 mg; VB_1_, 4 mg; VB_2_, 8 mg; VB_6_, 4.8 mg; VB_12_, 0.016 mg; nicotinic acid, 28 mg; pantothenic acid calcium, 16 mg; biotin, 0.064 mg; folic acid, 1.285 mg, inositol, 40 mg. ^2^ One kilogram of diet provided: Ca, 1150 mg; K, 180 mg; Mg, 45 mg; Fe, 50 mg; Zn, 40 mg; Mn, 9.5 mg; Cu, 7.5 mg; Co, 1.25 mg; I, 0.16 mg; Se, 0.25 mg.

## Data Availability

Data are available upon request, please contact the contributing authors.
